# Efficacy of subcutaneous doses and a new oral amorphous solid dispersion formulation of flubendazole on male jirds (*Meriones unguiculatus*) infected with the filarial nematode *Brugia pahangi*

**DOI:** 10.1371/journal.pntd.0006787

**Published:** 2019-01-16

**Authors:** Chelsea Fischer, Iosune Ibiricu Urriza, Christina A. Bulman, KC Lim, Jiri Gut, Sophie Lachau-Durand, Marc Engelen, Ludo Quirynen, Fetene Tekle, Benny Baeten, Brenda Beerntsen, Sara Lustigman, Judy Sakanari

**Affiliations:** 1 Dept. of Pharmaceutical Chemistry, University of California San Francisco, San Francisco, California, United States of America; 2 Janssen R&D, Janssen Pharmaceutica, Beerse, Belgium; 3 Veterinary Pathobiology, University of Missouri-Columbia, Columbia, Missouri, United States of America; 4 Laboratory of Molecular Parasitology, Lindsley F. Kimball Research Institute, New York Blood Center, New York, New York, United States of America; McGill University, CANADA

## Abstract

River blindness and lymphatic filariasis are two filarial diseases that globally affect millions of people mostly in impoverished countries. Current mass drug administration programs rely on drugs that primarily target the microfilariae, which are released from adult female worms. The female worms can live for several years, releasing millions of microfilariae throughout the course of infection. Thus, to stop transmission of infection and shorten the time to elimination of these diseases, a safe and effective drug that kills the adult stage is needed. The benzimidazole anthelmintic flubendazole (FBZ) is 100% efficacious as a macrofilaricide in experimental filarial rodent models but it must be administered subcutaneously (SC) due to its low oral bioavailability. Studies were undertaken to assess the efficacy of a new oral amorphous solid dispersion (ASD) formulation of FBZ on *Brugia pahangi* infected jirds (*Meriones unguiculatus*) and compare it to a single or multiple doses of FBZ given subcutaneously. Results showed that worm burden was not significantly decreased in animals given oral doses of ASD FBZ (0.2–15 mg/kg). Regardless, doses as low as 1.5 mg/kg caused extensive ultrastructural damage to developing embryos and microfilariae (mf). SC injections of FBZ in suspension (10 mg/kg) given for 5 days however, eliminated all worms in all animals, and a single SC injection reduced worm burden by 63% compared to the control group. In summary, oral doses of ASD formulated FBZ did not significantly reduce total worm burden but longer treatments, extended takedown times or a second dosing regimen, may decrease female fecundity and the number of mf shed by female worms.

## Author summary

Safe and effective macrofilaricidal drugs are critically needed to treat onchocerciasis and lymphatic filariasis, which affect over 54 million people worldwide. Flubendazole (FBZ) in its current commercial formulations is an effective anthelminthic for intestinal soil transmitted helminth (STH) infections but not for filarial infections due to its low bioavailability. The purpose of this study was to assess the efficacy of a new amorphous solid dispersion (ASD) formulation of FBZ given orally to jirds (*Meriones unguiculatus*) infected with the filarial nematode *Brugia pahangi* and compare it to FBZ (in suspension) given subcutaneously as a single or multiple dose. Our results indicated that treatment with ASD FBZ did not significantly reduce the total number of worms. However, doses as low as 1.5 mg/kg caused ultrastructural damage to the early stages of developing embryos. No worms were recovered from jirds given 10 mg/kg SC injections of FBZ (suspension) for 5 days when necropsied 68 days after the first-dose, and the number of mf at necropsy was significantly decreased compared to the control group. Animals given only a single 10 mg/kg SC injection had a 63% decrease in the number of adult worms. These animals also had fewer female worms and mf compared to the control group suggesting that even a single SC injection of FBZ had an effect on female survival and fecundity. TEM of worms recovered from jirds given low doses of the ASD formulated FBZ (1.5 mg/kg) suggested that female fecundity and mf production were reduced, but longer treatments, longer takedown times or a second dosing regimen of ASD FBZ may be needed to significantly decrease the total worm burden.

## Introduction

River blindness (onchocerciasis) and lymphatic filariasis are two major neglected diseases caused by parasitic nematodes that together affect an estimated 54 million people worldwide in mostly poor, developing countries.

With river blindness, approximately 12 million people suffer from skin disease and 1 million people have vision loss [1]. It is a chronic disease caused by the first larval stage, microfilariae (mf) of *Onchocerca volvulus* which are released from female worms residing in subcutaneous tissues. Microfilariae migrate throughout the skin causing severe itchiness, and in the eye, they induce an inflammatory response that eventually leads to blindness [2–5].

Lymphatic filariasis (LF) or elephantiasis is caused by *Wuchereria bancrofti*, *Brugia malayi* and *B*. *timori* whose adult worms infect and damage the lymphatic tissues. This debilitating disease is characterized by pain and severe lymphedema often involving the arms, legs, breasts and genitalia, leading to great economic losses as well as social suffering and the stigma associated with elephantiasis [2, 6].

To date, there are no vaccines for these diseases and international control programs attempt to interrupt transmission of infection in Africa with mass drug administration (MDA) annually or biannually using microfilaricidal drugs: ivermectin for onchocerciasis; albendazole plus ivermectin or albendazole plus diethylcarbamazine (DEC) for LF. Recently a triple-drug therapy with ivermectin, DEC and albendazole was explored for the treatment of LF outside of Africa with the assumption that it will accelerate the elimination of LF if a coverage of >65% of the population is achieved [7]. Notably, this new therapy significantly improves mf clearance and maintenance of amicrofilaremia compared to the two-drug MDA regimen with DEC and albendazole and offers great promise in eliminating LF [7, 8].

The triple-drug therapy however, is not relevant for treatment of onchocerciasis due to the major adverse affects caused by DEC [9]. Although elimination of onchocerciasis has been achieved in a few foci in Africa and in the Americas [10–12], there has been only a 31% reduction in the incidence of onchocerciasis in Africa since 1995 [13]. The African Program for Onchocerciasis Control (APOC) has therefore called for some 1.15 billion treatments by 2045 [14], though many neglected tropical disease experts doubt that onchocerciasis can ever be eliminated through MDA alone [15], especially given that MDA with ivermectin cannot be used in 11 Central African countries co-endemic with *Loa loa* infections due to the risk of severe adverse events [16, 17]. Given the longevity and high fecundity of the adult worms (macrofilariae) [18–22] and the current lack of macrofilaricidal drugs, it is unlikely that the WHO goal of eliminating onchocerciasis by 2025 will be met when microfilaricidal drugs alone are used [17, 23, 24].

To achieve the ultimate goal of onchocerciasis elimination, drugs that cure infections and thus stop transmission of infection and ultimately shorten the time to elimination, a safe and effective drug that kills adult worms is needed. The need for such alternate treatment strategies is further supported by the occurrence of foci in Africa with suboptimal response to ivermectin [25].

The benzimidazole anthelmintic flubendazole (FBZ) is highly efficacious as a macrofilaricide in experimental filarial rodent models but it must be administered subcutaneously (SC) due to its low oral bioavailability [26–28]. Unfortunately, when administered parenterally to patients with onchocerciasis, severe reactions around the intramuscular injection site were reported [9]. Therefore, efforts have been made to develop a re-formulation of FBZ that would enable oral dosing [26–28]. The purpose of this present study was to assess the efficacy of a new amorphous solid dispersion formulation of FBZ (ASD FBZ; Janssen Pharmaceutica) for the treatment of onchocerciasis. ASD FBZ was given orally (0.2, 0.6, 1.5, 6, 15 mg/kg for 5 days) to jirds (*Meriones unguiculatus*) infected with the filarial nematode *Brugia pahangi* and compared to a single and a 5 day SC injection of 10 mg/kg FBZ. The rodent model using jirds as hosts for adult *B*. *pahangi* has been used extensively to study efficacy of antifilarial compounds and is one of the surrogate models used to investigate drugs for treatment of onchocerciasis [26, 29–33]. Results of this study showed that worm burden was not significantly decreased in jirds given oral doses of ASD FBZ (0.2–15 mg/kg). However, doses as low as 1.5 mg/kg caused extensive ultrastructural damage to developing embryos and mf.

## Methods

### Experimental infection

Male jirds (*Meriones unguiculatus*) approximately 6 weeks of age (50–60 g) were purchased from Charles River (USA, Kingston K62 jirds) and infected by intraperitoneal injection with *Brugia pahangi* third-stage larvae (L3). Dosing regimens began a minimum of 12 weeks post-infection following development of the larval stage to the adult stages and secretion of microfilariae. Animals were allowed to eat and drink *ad libitum* and maintained following the approved IACUC protocol AN109629-03D.

### Formulations

Oral suspensions of ASD flubendazole (Janssen Bend 1/9) contained 10% FBZ-AAA G001, 10% flubendazole:hydroxypropyl methylcellulose acetate succinate, Lot number BREC-1113-036 with a vehicle of aqueous solution of 0.5% w/v methocel A4M (Premium) in demineralized water. For the flubendazole subcutaneous suspension (FBZ-AAA, lot 0020470007), FBZ was purchased from Shaanxi Hanjiang Pharmaceutical Group LTD, Hanzhong City, Shaanxi, China and formulated with aqueous solution of 0.5% w/v HEC (2-hydroxyethyl cellulose, Sigma 434965) in demineralized water and 0.1% Tween 80. Formulations were acclimated for 30 min at room temperature, protected from light and homogenized prior to dosing for at least 30 seconds to ensure no visible sedimentation.

### Dosage groups

ASD flubendazole was given per os (PO) at 0.2, 0.6, 1.5, 6 or 15 mg/kg for 5 consecutive days, or subcutaneously (SC) at 10 mg/kg one time or for 5 days; control groups were not given any treatment which allowed comparison with both the oral and SC groups rather than having to include 2 different vehicle groups (Table 1). Doses were selected to determine the efficacy of the new ASD formulation of FBZ after oral administration for 5 days, which is considered a dosing regimen feasible for use in patients in the field as well as on the basis of previous pharmacokinetic and toxicological data. For the subcutaneous route and doses, the 5-day 10 mg/kg dose group is used as a positive control group in antifilarial rodent models. A single dose subcutaneous dose of 10 mg/kg was included to test if the same efficacy could be obtained as with the positive control group.

**Table 1:** Dosing regimens for ASD FBZ studies.

EXPT	Formulation	n =	Route of administration	mg/kg	# days of dosing
**1**	FBZ suspension	7	SC	10	1
	FBZ suspension	6	SC	10	5
	ASD FBZ	7	PO	0.2	5
	ASD FBZ	7	PO	0.6	5
	ASD FBZ	7	PO	1.5	5
	No treatment control	6			
**2**	FBZ suspension	10	SC	10	1
	ASD FBZ	10	PO	6	5
	ASD FBZ	9	PO	15	5
	No treatment control	10			

All animals used in the study were lightly anesthetized with isoflurane just to the state of drowsiness prior to dosing to avoid any handling stress. Animals were given PO doses using a metal gavage needle and 1 ml tuberculin syringe. Animals receiving SC doses were injected on the scruff of their necks with a 25 gauge needle in a clockwise fashion to avoid injection into the same site over the 5 day treatment period. The untreated control group was also given light anesthesia as in the case with the treated animals but was not dosed. Animals were allowed to feed ad libitum and dosed according to their body weight determined prior to each dosing. The takedown times were 68 and 72 days post-first dose.

### Samples for pharmacokinetics (PK) of ASD FBZ

The sparse sampling approach was used to obtain a sufficient time profile while minimizing stress to the animals, e.g. 2–3 animals per group were sampled per time point using the micro-sampling technique. Blood sampling times were chosen based on the route and duration of treatment and previous PK data, and thereby took into account the allowable volume of blood that could be taken from gerbils without causing stressful manipulation of the animals. Approximately 30–50 μl of blood was collected per animal from the vena saphena, using heparin-coated hematocrit capillary tubes. Blood was placed immediately on ice and centrifuged for 1810 g for 15 min at 4°C. 10 μl of plasma was then transferred into a PCR microfuge tube, frozen in a dry ice/ethanol bath and stored at -80°C until shipped.

Plasma samples from jirds were collected at the following times for Experiment 1: For the SC single dose (10 mg/kg): 1, 3, 8 and 24 hrs post-dose; for the SC repeat doses (10 mg/kg): 2 hrs post-dose on Days 1–4; at Day 5: 1, 3, and 8, 24, 48 hrs post-dose; for the SC groups: weekly for 9 weeks post-last dose; for the PO repeat doses: 2 hrs post-dose on Days 1–4; 2 hrs and 24 hrs post-dose on Days 5. Plasma samples from jirds were collected at the following times for Experiment 2: for the SC single dose (10 mg/kg): 1, 3, 8, 24 and 48 hrs post-dose; and 1, 3, 5, 7, and 9 weeks post-dose; for the PO repeat doses (6 mg/kg and 15 mg/kg): 2 hrs post-dose on Days 1–4; 1, 2, 4, 8 and 24 hrs post-dose on Day 5. All flubendazole formulations used in the study were also analyzed at the end of the dosing period.

For Experiment 1, analyses were conducted at Janssen Research and Development (1400 McKean Road, Spring House, PA 19477), and for Experiment 2, analyses were conducted at Janssen Research and Development, Beerse, Belgium. For both studies, plasma samples were analyzed individually for flubendazole (JNJ-161941), hydrolyzed flubendazole (H-FBZ, JNJ-114699) and reduced flubendazole (R-FBZ, JNJ-1809600) using a qualified LC-MS/MS method. 10 μl plasma aliquots in end-to-end capillaries were washed with 100μl of 2% BSA in phosphate buffer pH 7.5. From this diluted sample 44 μl was taken for analysis corresponding to 4 μl of plasma. After addition of 10 μl of internal standard dilution and 200 μl of acetonitrile for the precipitation of the plasma proteins, the samples were mixed and centrifuged. 150 μl of the supernatant was evaporated to dryness under nitrogen flow at 50°C and reconstituted in 150 μl of a mixture of 0.1% formic acid and acetonitrile (90/10, v/v). 20 μl of the extract was injected onto an Acquity UPLC BEH C18 column (50 x 2.1 mm, 1.7μm particles) (Waters, Milford, USA). The chromatographic system consisted of a Shimadzu SIL30ACMP autosampler and Shimadzu LC30 pumps (Shimadzu, Kyoto, Japan). The mobile phase was a mixture of 1% formic acid and acetonitrile with a flow rate of 0.6 ml/min and a 2.5 minute gradient from 20 to 60% acetonitrile followed by a 1-minute step gradient to 98% acetonitrile.

Mass spectrometric detection was performed on an API4000 triple quadrupole mass spectrometer (Sciex, Framingham, MA, USA) with Turbo Ion Spray^TM^ ionization operated in positive ion mode. FBZ, H-FBZ and R-FBZ were quantified against calibration samples and quality control samples, prepared in the same matrix as the study samples by means of a qualified analytical method with a lower limit of quantitation of 0.2, 0.4 and 0.2 ng/ml, respectively and an upper limit of quantitation of 3000 ng/ml for all three analytes.

### Animal necropsies

Animals were euthanized on day 68 (Expt 1) and day 72 (Expt 2) after the first dose. Adult worms and mf were recovered by opening the body cavity and washing the peritoneal cavity with 100 mL of phosphate buffered saline (PBS). Male and female worms were separated and counted using a dissecting microscope. To count the number of mf present in the peritoneal cavity at necropsy, a 100 μl sample of the aforementioned PBS washing fluid was added to 900 μl of 0.04% methylene blue, and then a 50–150 μl sample of the stained mf was streaked onto a glass slide and counted using a compound microscope. The sample mf counts were multiplied by the appropriate dilution factor to calculate the total number of mf from each jird.

### Ethics statement

Animals were euthanized by carbon dioxide inhalation followed by bilateral thoracotomy, following University of California San Francisco IACUC protocol AN109629-03D.

### Transmission electron microscopy (TEM) of female *Brugia pahangi*

At necropsy, 7–12 female worms recovered from 2–3 jirds per group were fixed in 2.5% paraformaldehyde, 2% glutaraldehyde in 0.1 M cacodylate buffer, pH 7.4 [34, 35]. Worms were chopped into 1–2 mm long pieces in the fixative. Samples were incubated for 3 hrs at room temperature protected from light and kept at 4°C overnight. Samples were then washed thoroughly in buffer and post-fixed in 1% osmium tetroxide and 1.6% potassium ferricyanide in 0.1 M cacodylate buffer, pH 7.4, for 1 hr. Following washes in buffer and then in distilled water, en bloc staining was performed for 1 hr with 2% uranyl acetate with samples protected from light. Samples were again washed with water and dehydrated through a series of ethanol dilutions: 50%, 70%, 95% and 100% ethanol. Samples were infiltrated with a gradient of acetone-Embed 812 resin and embedded in 100% resin. After sectioning the solidified blocks, 70 nm sections were post-stained with 1% uranyl acetate and Reynolds lead citrate. Images of the sections were collected on a 120kV Microscope (Philips).

### Statistical analyses

To compare adult worm and mf counts in the treated groups versus the control group, raw data were first tested for normality using the Shapiro-Wilk test. When data did not pass the Shapiro-Wilk test, data were then log_10_ transformed and retested using the Shapiro-Wilk test. In Experiment 2 the log_10_ transformation of the total number of adult worms per jird passed the Shapiro-Wilk test, so significance was determined by a one-way ANOVA followed by the Holm-Sidak multiple comparisons test. The remaining data did not pass the Shapiro-Wilk test, even after log_10_ transformation, so significance was determined by the Kruskal-Wallis test, followed by Dunn’s multiple comparison test. All data were analyzed using Prism 6.0f 2014, GraphPad Software, Inc. with 95% confidence limits.

To calculate the geometric means of the number of adult worms, female worms and mf recovered at necropsy, 0.1 was used in place of 0. The percent efficacy was calculated for each treatment group by subtracting the geometric means of treatment groups from the geometric mean of the control group, multiplying by 100%, and dividing the numerator by the geometric mean of the control group. All results written as percentages are given as a geometric mean % (e.g. geometric mean % reduction).

## Results

### Repeat SC injections of FBZ in suspension are effective in decreasing worm burden

**Fig 1:**
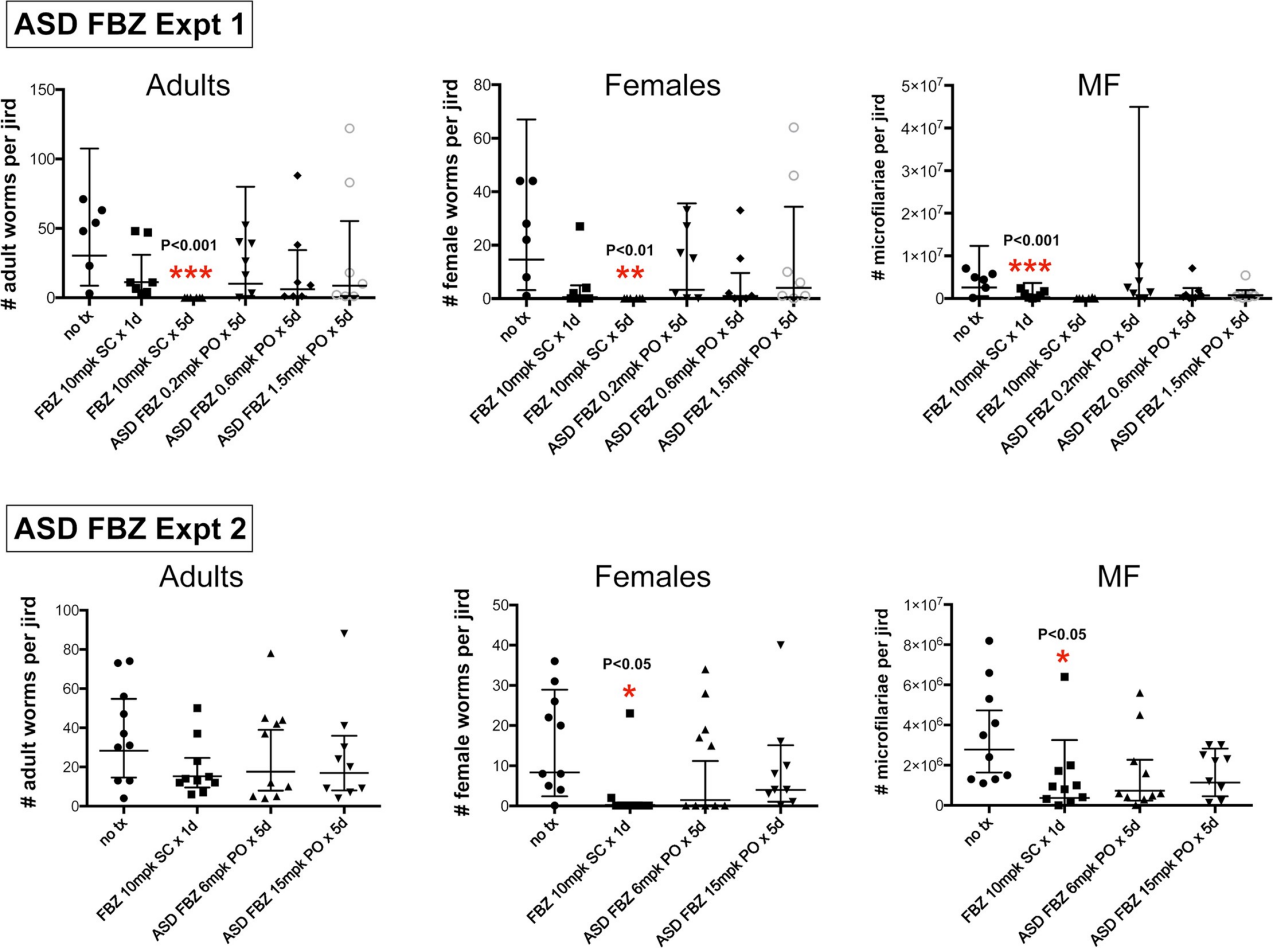
Scatter plots from Expt 1 and Expt 2 of the total number of worms, female worms and mf recovered from each jird per group (geometric means ± 95% confidence limits).

No significant pathology occurred at the site of injection nor was any pathology observed over the course of the experimental period when SC doses of FBZ were injected into the nape area of the jirds. No adult worms were recovered from animals given 10 mg/kg SC injections of FBZ for 5 days, and the number of mf was significantly decreased compared to the control group (Fig 1 and Table 2). In comparison, animals given a single SC injection of 10 mg/kg FBZ had only a 63% (not statistically significant) decrease in the number of adult worms compared to the control group. These animals however, had fewer female worms and mf suggesting that even a single SC injection had an effect on female survival and fecundity. This effect was also observed in Experiment 2 for animals given a single SC injection of FBZ (Fig 1 and Table 2).

**Table 2:** Summary of efficacy results of jirds infected with *Brugia pahangi* treated with ASD FBZ.

EXPT	Treatment	% reduction adult worms (geometric means)	% reduction female adult worms (geometric means)	% reduction mf (geometric means)
**1**	SC 10 mg/kg x 1 day	63	96	97
	SC 10 mg/kg x 5 days	100	100	100
	PO 0.2 mg/kg x 5days	67	77	96
	PO 0.6 mg/kg x 5 days	80	93	73
	PO 1.5 mg/kg x 5 days	72	72	71
**2**	SC 10 mg/kg x 1 day	46	97	87
	PO 6 mg/kg 5 days	38	82	74
	PO 15 mg/kg 5 days	40	52	59

### Oral doses of ASD FBZ did not significantly reduce female worm recovery nor number of mf

There was no statistically significant reduction in the total number of adult *Brugia pahangi* recovered from jirds given oral doses of ASD FBZ compared to the control group (Fig 1 and Table 2). Reduction in the total number of worms ranged from 38% to 80% and did not correlate with ASD FBZ dose concentrations. There was also no statistical difference in the percent reduction in the number of female worms nor mf in the treatment groups compared to the control group due to the high variability within each group. However, the results showed that all groups given oral ASD FBZ still had a 52%-93% reduction in the number of female worms recovered at necropsy and 59%-96% reduction in the mf count, respectively (Fig 1 and S1 Table).

### PK results for ASD FBZ studies

**Fig 2:**
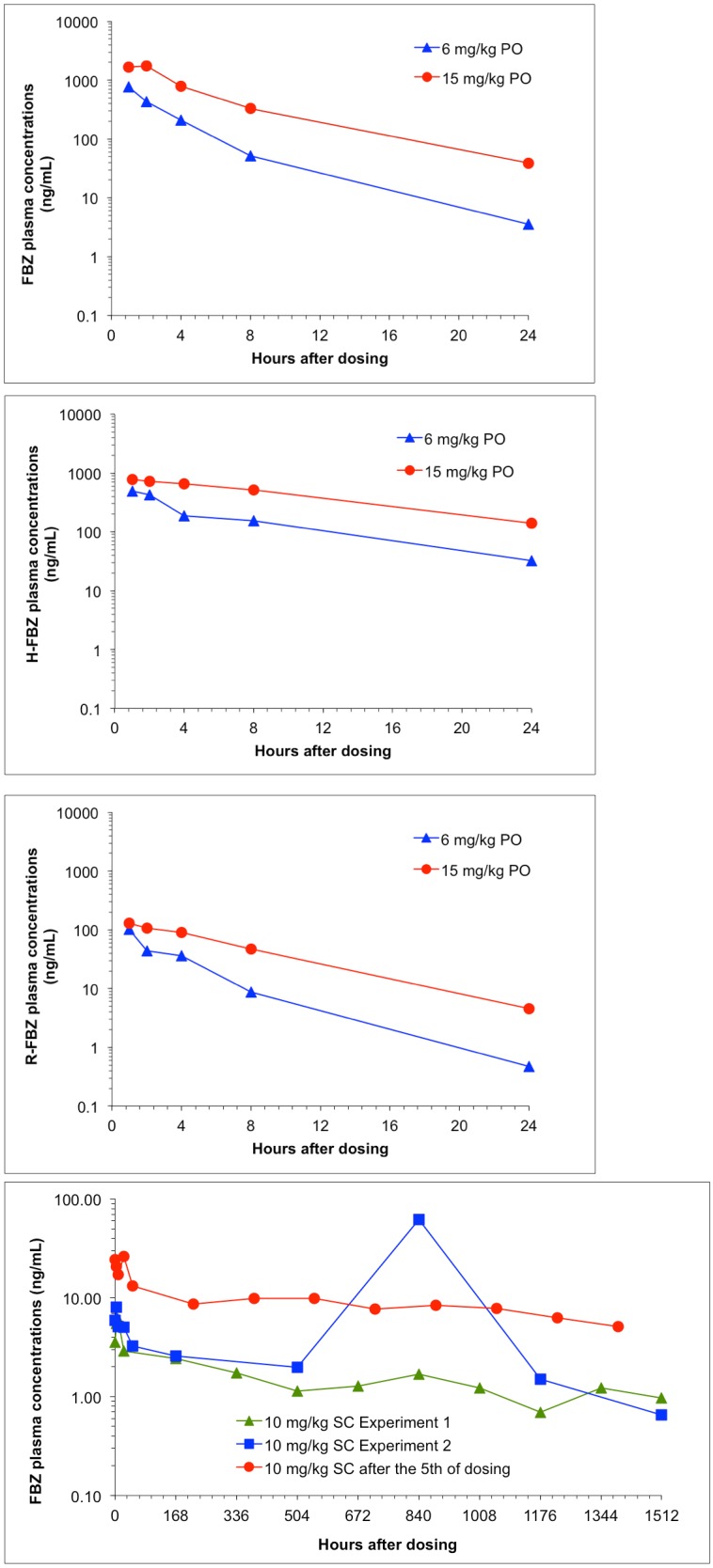
Plasma concentrations from male jirds given 6 and 15 mg/kg ASD FBZ orally for 5 days (FBZ, H-FBZ and R-FBZ) and subcutaneously (SC) at 10 mg/kg, once and for 5 days.

Individual and mean plasma concentrations and pharmacokinetic (PK) parameters were determined for FBZ and the metabolites, H-FBZ and R-FBZ (Tables 3–6, Fig 2, S2 Table). Results showed that after oral administration, concentrations of FBZ from the 2 hr plasma samples increased with the dose (Table 3). At day 5, the mean concentration at 2 hr post-dose was 0.015 μg/ml for 0.2 mg/kg, 0.039 μg/ml for 0.6 mg/kg, 0.085 μg/ml for 1.5 mg/kg, 0.436 μg/ml for 6 mg/kg and 1.73 μg/ml for 15 mg/kg. At 24 hr on day 5, the plasma concentrations were similar between 0.2 and 0.6 mg/kg/ day and increased with the dose between 0.6 and 15 mg/kg/day.

**Table 3:** Mean (± s.d) plasma concentrations at 2 hr after PO dosing of ASD FBZ with 0.2, 0.6, 1.5, 6 and 15 mg/kg/day.

PO						
Doses (mg/kg/day)	Concentrations of FBZ at 2 hours after dosing (ng/ml)					Concentrations at 24h
	Day 1	Day 2	Day 3	Day 4	Day 5	Day 5
0.2^a^	7.43 ± 5.92	9.77 ± 5.46	10.2 ± 2.6	14.2 ± 10.3	14.6 ± 0.7	0.559 ± 0.391
0.6^a^	21.8 ± 16.8	22.4 ± 9.6	35.8 ± 30.7	29.1 ± 21.7	38.5 ± 10.9	0.547 ± 0.223
1.5^a^	68.9 ± 24.2	79.5 ± 43.0	56.8 ± 8.7	70.4 ± 35.5	84.6 ± 42.1	1.38 ± 1.06
6^b^	268	464	368	424	436	3.54
15^b^	1562	2084	2498	974	1726	39.1

^a^: n = 3

^b^: n = 2; s.d: standard deviation

**Table 4:** PK parameters of ASD FBZ at the last day of dosing after repeated oral administration for 5 days. AUC (Area Under the Curve) is total drug exposure in blood plasma against time and C_max_ is highest observed blood plasma concentration.

PO					
Doses (mg/kg/day)	C_max_ (ng/mL)	t_max_ (h)	AUC_0-24h_ (ng•h/mL)	H-FBZ /FBZ AUC ratio	R-FBZ/FBZ AUC ratio
6	768	1	2323	1.4	0.14
15	1726	2	9202	1.0	0.10

C_max_: highest observed blood plasma concentration and t_max_: time point when C_max_ is observed

**Table 5:** PK parameters of FBZ after a single SC dose for experiment 1 and experiment 2.

SC						
Experiment	Doses (mg/kg) 1 day	C_max_ (ng/mL)	t_max_ (h)	AUC_0-1512h_ (ng•h/mL)	H-FBZ /FBZ AUC ratio	R-FBZ/FBZ AUC ratio
1	10	6.07	8	2270	0.96	0.18
2	10	62.2	840	17941	0.2	0.001

**Table 6:** PK parameters of FBZ after the last day of dosing after repeated subcutaneous administration for 5 days.

SC						
Experiment	Doses (mg/kg) x 5 days	C_max_ (ng/mL)	t_max_ (h)	AUC_0-1392h_ (ng•h/mL)	H-FBZ /FBZ AUC ratio	R-FBZ/FBZ AUC ratio
1	10	26.1	24	12280	1.4	0.17

FBZ exposure (AUC_0-24h_) at day 5 increased more than dose proportionally to the dose between 6 and 15 mg/kg/day (Table 4). C_max_ however was dose proportional. The t_max_ for repeat oral administration of FBZ at 6 and 15 mg/kg/day for 5 days was 1 hr and 2 hr, respectively, indicating fast absorption. Ratios of H-FBZ/FBZ AUC were 10-fold higher than for R-FBZ/FBZ AUC (Fig 2, Table 4).

For the SC groups, the following PK parameters were calculated: 10 mg/kg SC single dose (Table 5, Fig 2): AUC_0-last_ = 2.3 μg•hr/mL with t _last_ at 1512 hr; C_max_ = 0.06 μg/mL at 8 hr. For the 10 mg/kg SC dose repeated for 5 days (Table 6): AUC_0-last on day 5_ = 15 μg•hr/mL with t _last_ at 1512 hr; C_max_ = 0.26 μg/mL at 24 hr. After a single subcutaneous administration of FBZ at 10 mg/kg, peak plasma concentrations after dosing were observed at 8 hr in the first experiment and 840 hr in the second experiment (Table 5). FBZ was measured up to the last sampling time point (1512h).

After repeated subcutaneous administration of FBZ at 10 mg/kg for 5 days, peak plasma concentrations were observed at 24 hr after the last dosing (Fig 2, Table 6). FBZ was measured up to the last sampling time point (1392 h). The H-FBZ/FBZ AUC ratio ranged from 0.2 to 1.4 and ranged from 0.001 to 0.18 for R-FBZ/FBZ AUC ratio across all FBZ dosed groups (Tables 4–6).

### Transmission electron microscopic analyses of female worms recovered from ASD FBZ

**Fig 3:**
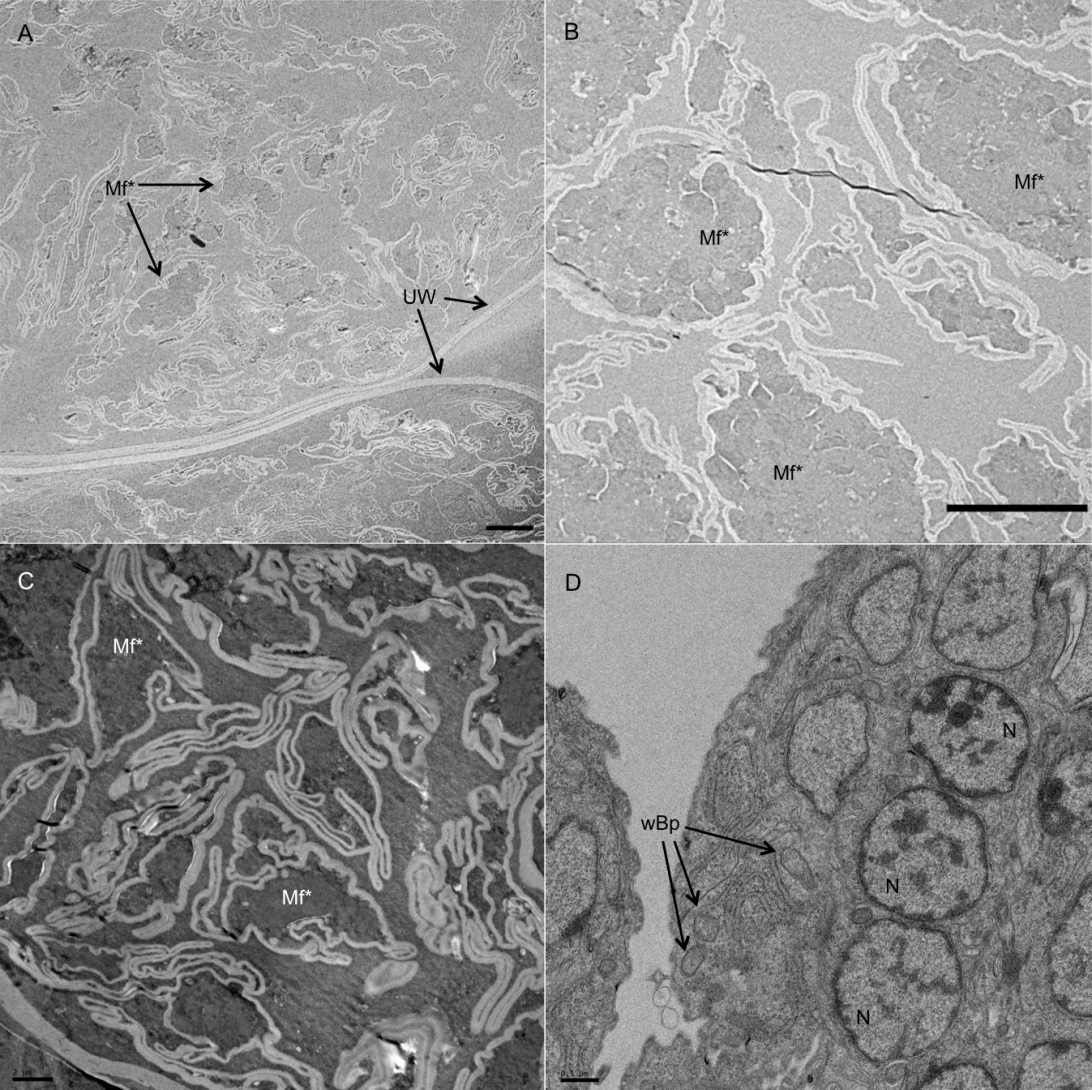
Transmission electron micrographs of uteri from female worms collected from jirds treated orally with 1.5 mg/kg ASD FBZ for 5 days. (A) Lower magnification showing the damage throughout the uterine tissues. (B) Higher magnification of damaged microfilariae (Mf*) showing no nuclei or structural integrity (scale bars = 5 μm). (C) Microfilariae within a female worm from an animal treated SC with a single injection of 10 mg/kg FBZ showed similar damage as that seen in worms treated with 1.5 mg/kg ASD FBZ for 5 days. (D) Section of microfilariae within a female worm from a control animal showed normal structural morphology (scale bar = 0.5 μm). UW–uterine wall; Mf*–degraded microfilariae; wBp–*Wolbachia* of *Brugia pahangi*; N–nucleus.

Although the treatment with the highest oral dose of ASD FBZ (15 mg/kg) did not result in a significant reduction in worm burden, the ultrastructural analysis of female worms recovered from jirds dosed with 1.5 mg/kg ASD FBZ for 5 days revealed extensive morphological alteration in embryos and developing mf. This observation is similar to what was seen in worms treated with a single SC injection of 10 mg/kg FBZ (Fig 3). Eggshells surrounding embryos were disrupted and disorganized compared to eggshells of developing embryos within the gonads of female worms recovered from control animals.

## Discussion

The benzimidazole anthelmintic flubendazole (FBZ) had been shown to be an excellent macrofilaricidal drug but its use was limited because of its need to be administered subcutaneously due to its low oral bioavailability [26] and adverse reactions at the site of injection [9]. Although efforts were made to reformulate FBZ as an oral drug [26–28], it did not move to clinical development. The purpose of the present study was to assess the efficacy of a new amorphous solid dispersion oral formulation of FBZ (ASD FBZ). ASD FBZ was administered orally to *Brugia pahangi* infected jirds at doses of 0.2, 0.6, 1.5, 6 and 15 mg/kg for 5 days to assess the effects of this new formulation of FBZ on adult worm burden, number of female worms and microfilarial counts. Although there was no statistically significant decrease in total number of worms, female worms or mf recovered from treated animals compared to untreated animals, ASD FBZ appeared to have some effect on the fitness of female worms and their fecundity as evidenced by the relatively lower number of female worms (60–88% reduction) and mf (59–96% reduction). This may suggest that ASD FBZ had an effect on female worm viability, which in turn, caused the reduction in the number of mf that were shed.

Hübner et al. observed histological damage in female *Litomosoides sigmodontis* from jirds given oral ASD FBZ doses of 6 and 15 mg/kg for 5 days and with 2 and 6 mg/kg for 10 days [32]. Degenerative changes were seen in the body wall, intestine and uteri, with major structural damage to the developing mf. In the present study, TEMs of ovaries and uteri from *B*. *pahangi* females recovered from jirds treated with 1.5 mg/kg ASD FBZ for 5 days showed that this treatment also caused extensive damage to the developing mf. Thus ASD FBZ appears to cause similar effects in the *Brugia*/jird model as in the *Litomosoides*/jird model.

TEMS also revealed extensive ultrastructural damage to mf from female worms retrieved from animals given only a single SC injection of FBZ suggesting that a low dose of orally administered ASD FBZ given for 5 days may cause damage to developing mf, similar to when animals are given a single 10 mg/kg FBZ injection. In a study by Franz et al., a single SC dose of 25 mg/kg of FBZ impaired cell division in oogonia and embryonic cells in *B*. *malayi* female worms [36]. The damage to developing mf and female fecundity may account for the reduction in the number of mf (albeit not statistically significant reduction) seen in treated jirds in the present study.

With the exception of one jird, all animals treated with oral ASD FBZ had mf at the time of necropsy which suggests ASD FBZ may have inhibited embryogenesis but it did not have a direct-acting effect on the mf that were already shed. *In vitro* studies by O’Neill et al. showed that concentrations as low as 100 nM of commercially purchased FBZ induced damage to the early embryonic developmental stages of *B*. *malayi* but caused little to no damage to the later stages (pretzel and stretched mf) in female worms cultured for 3 days [37]. Similar effects were observed when *B*. *malayi* adult worms were first incubated in 100 nM FBZ *in vitro* for 24 hr and subsequently implanted intraperitoneally into jirds. Female worms removed from jirds 8 weeks later contained fewer embryos, a larger number of degenerating embryos and released fewer mf compared to the controls [38]. Furthermore, a study by Sjoberg et al. found that ASD FBZ given orally to SCID mice at a dosage of 2 or 40 mg/kg was not directly microfilaricidal to *B*. *malayi* mf circulating in the blood for 2 days. They concluded that elevated exposures would not likely cause rapid killing of bloodborne mf, a major concern due to the severe adverse events occurring in individuals who had been treated with the microfilaricidal drug ivermectin while infected with high numbers of *Loa loa* mf [33].

In the present study, only 2 of the 6 animals from the group given SC injections of FBZ for 5 days had mf at the time of takedown. In this group, no female worms (nor male worms) were recovered from any of the jirds at necropsy. Since ASD FBZ does not appear to be microfilaricidal, the lower number of mf that were found in this group compared to the control group, were not due to any direct-acting effect but rather due to the elimination of female worms early in the dosing period.

The PK analyses indicated that FBZ given SC led to a slow FBZ release from the injection site and the low plasma levels remained constant up to the study endpoint. In contrast, after oral administration of ASD FBZ, higher plasma concentrations were observed with a rapid decline after the C_max_. Thus, it appears that the slowly released and sustained levels of FBZ following repeated SC injections for 5 days were highly effective in eliminating worms while the levels of ASD FBZ at the dosages given, were not effective in reducing the adult worm burden, number of female worms or mf.

The ASD formulation was selected based on data obtained in rats after testing at least 8 formulations in order to improve the bioavailability of the drug [39]. The selection of formulation took into account the feasibility and stability of the formulation and exposures. The ASD formulation was also tested in another study with female jirds infected with *Litomosoides sigmodontis* at the same oral doses of 6 and 15 mg/kg for 5 days [32]. At Day 5, the exposures in the present study were 2–3 fold lower than the Hübner study. Compared to an aqueous hydroxypropyl—β cyclodextrin solution that was administered to jirds at 5 mg/kg in a study by Ceballos et al. the exposure in the present study was slightly lower, most likely because the stability of FBZ in hydroxypropyl—β cyclodextrin solution was not optimal [27]. Flubendazole exposures are quite different across species, and this difference needs to be taken into consideration since both R-FBZ and H-FBZ may be potentially active in humans [40, 41].

Because Mongolian jirds are capable of maintaining *Brugia pahangi* infections for approximately 2 years, long-term efficacy studies should also be considered when evaluating macrofilaricidal drugs that may require long exposures. ASD FBZ given orally for 5 days did not significantly reduce worm burden but embryogenesis appears to have been affected at a dose as low as 1.5 mg/kg as evidenced by the damage to developing mf at the ultrastructural level. An extended takedown time beyond the 2-month time period may be required to observe a significant reduction in total number of worms and female fecundity or viability. Future studies should include longer takedown times with embryogram analyses to further substantiate the effects on female worm sterilization and fecundity when evaluating oral ASD FBZ as a macrofilaricidal drug.

## Supporting information

S1 Table Adult *B. pahangi* worm burden, number of female and microfilarial counts from individual male jirds (*Meriones unguiculatus*) for experiment 1 and experiment 2 with the geometric means ± 95% confidence limits and level of significance. Jirds received 10 mg/kg subcutaneous (SC) flubendazole injections once or for 5 days or oral (PO) doses of 0.2, 0.6, 1.5, 6 or 15 mg/kg flubendazole for 5 consecutive days.
(PDF)

S2 Table Plasma (ng/ml) concentrations of JNJ-161941 (FBZ) and the metabolites JNJ-114699 (H-FBZ) and JNJ-1809600 (R-FBZ) after oral (PO) and single or repeated subcutaneous (SC) administration to male jirds (n = 2 or 3 per time point). (PDF)

## References

[1] Global, regional, and national incidence, prevalence, and years lived with disability for 310 diseases and injuries, 1990–2015: a systematic analysis for the Global Burden of Disease Study 2015. Lancet. 2016;388(10053):1545–602. Epub 2016/10/14. 10.1016/S0140-6736(16)31678-6 27733282 PMC5055577

[2] Taylor MJ, Hoerauf A, Bockarie M. Lymphatic filariasis and onchocerciasis. Lancet. 2010;376(9747):1175–85. Epub 2010/08/27. 10.1016/S0140-6736(10)60586-7 . 20739055

[3] Tamarozzi F, Halliday A, Gentil K, Hoerauf A, Pearlman E, Taylor MJ. Onchocerciasis: the role of *Wolbachia* bacterial endosymbionts in parasite biology, disease pathogenesis, and treatment. Clin Microbiol Rev. 2011;24(3):459–68. Epub 2011/07/08. 10.1128/CMR.00057-10 21734243 PMC3131055

[4] Turner JD, Langley RS, Johnston KL, Gentil K, Ford L, Wu B, et al *Wolbachia* lipoprotein stimulates innate and adaptive immunity through Toll-like receptors 2 and 6 to induce disease manifestations of filariasis. J Biol Chem. 2009;284(33):22364–78. Epub 2009/05/22. 10.1074/jbc.M901528200 19458089 PMC2755959

[5] Pearlman E, Hall LR, Higgins AW, Bardenstein DS, Diaconu E, Hazlett FE, et al The role of eosinophils and neutrophils in helminth-induced keratitis. Invest Ophthalmol Vis Sci. 1998;39(7):1176–82. Epub 1998/06/10. . 9620077

[6] Murray CJ, Vos T, Lozano R, Naghavi M, Flaxman AD, Michaud C, et al Disability-adjusted life years (DALYs) for 291 diseases and injuries in 21 regions, 1990–2010: a systematic analysis for the Global Burden of Disease Study 2010. Lancet. 2012;380(9859):2197–223. Epub 2012/12/19. 10.1016/S0140-6736(12)61689-4 . 23245608

[7] Irvine MA, Stolk WA, Smith ME, Subramanian S, Singh BK, Weil GJ, et al Effectiveness of a triple-drug regimen for global elimination of lymphatic filariasis: a modelling study. Lancet Infect Dis. 2017;17(4):451–8. Epub 2016/12/26. 10.1016/S1473-3099(16)30467-4 . 28012943

[8] Thomsen EK, Sanuku N, Baea M, Satofan S, Maki E, Lombore B, et al Efficacy, safety, and pharmacokinetics of coadministered diethylcarbamazine, albendazole, and ivermectin for treatment of Bancroftian filariasis. Clin Infect Dis. 2016;62(3):334–41. Epub 2015/10/22. 10.1093/cid/civ882 . 26486704

[9] Dominguez-Vazquez A, Taylor HR, Greene BM, Ruvalcaba-Macias AM, Rivas-Alcala AR, Murphy RP, et al Comparison of flubendazole and diethylcarbamazine in treatment of onchocerciasis. Lancet. 1983;1(8317):139–43. Epub 1983/01/22. . 6130195 10.1016/s0140-6736(83)92753-8

[10] Diawara L, Traore MO, Badji A, Bissan Y, Doumbia K, Goita SF, et al Feasibility of onchocerciasis elimination with ivermectin treatment in endemic foci in Africa: first evidence from studies in Mali and Senegal. PLoS Negl Trop Dis. 2009;3(7):e497 Epub 2009/07/22. 10.1371/journal.pntd.0000497 19621091 PMC2710500

[11] Traore MO, Sarr MD, Badji A, Bissan Y, Diawara L, Doumbia K, et al Proof-of-principle of onchocerciasis elimination with ivermectin treatment in endemic foci in Africa: final results of a study in Mali and Senegal. PLoS Negl Trop Dis. 2012;6(9):e1825 Epub 2012/10/03. 10.1371/journal.pntd.0001825 23029586 PMC3441490

[12] Gustavsen K, Hopkins A, Sauerbrey M. Onchocerciasis in the Americas: from arrival to (near) elimination. Parasit Vectors. 2011;4:205 Epub 2011/10/26. 10.1186/1756-3305-4-205 22024050 PMC3214172

[13] Global, regional, and national incidence, prevalence, and years lived with disability for 301 acute and chronic diseases and injuries in 188 countries, 1990–2013: a systematic analysis for the Global Burden of Disease Study 2013. Lancet. 2015;386(9995):743–800. Epub 2015/06/13. 10.1016/S0140-6736(15)60692-4 26063472 PMC4561509

[14] Kim YE, Remme JH, Steinmann P, Stolk WA, Roungou JB, Tediosi F. Control, elimination, and eradication of river blindness: scenarios, timelines, and ivermectin treatment needs in Africa. PLoS Negl Trop Dis. 2015;9(4):e0003664 Epub 2015/04/11. 10.1371/journal.pntd.0003664 25860569 PMC4393239

[15] Keenan JD, Hotez PJ, Amza A, Stoller NE, Gaynor BD, Porco TC, et al Elimination and eradication of neglected tropical diseases with mass drug administrations: a survey of experts. PLoS Negl Trop Dis. 2013;7(12):e2562 Epub 2013/12/18. 10.1371/journal.pntd.0002562 24340111 PMC3855072

[16] Kelly-Hope LA, Cano J, Stanton MC, Bockarie MJ, Molyneux DH. Innovative tools for assessing risks for severe adverse events in areas of overlapping Loa loa and other filarial distributions: the application of micro-stratification mapping. Parasit Vectors. 2014;7:307 Epub 2014/07/06. 10.1186/1756-3305-7-307 24992829 PMC4101798

[17] Molyneux DH, Hopkins A, Bradley MH, Kelly-Hope LA. Multidimensional complexities of filariasis control in an era of large-scale mass drug administration programmes: a can of worms. Parasit Vectors. 2014;7:363 Epub 2014/08/17. 10.1186/1756-3305-7-363 25128408 PMC4261528

[18] Plaisier AP, van Oortmarssen GJ, Remme J, Habbema JD. The reproductive lifespan of *Onchocerca volvulus* in West African savanna. Acta Tropica. 1991;48(4):271–84. Epub 1991/02/01. . 1674401 10.1016/0001-706x(91)90015-c

[19] Hoerauf A, Pfarr K, Mand S, Debrah AY, Specht S. Filariasis in Africa—treatment challenges and prospects. Clin Microbiol Infect. 2011;17(7):977–85. Epub 2011/07/05. 10.1111/j.1469-0691.2011.03586.x . 21722251

[20] Lustigman S, Prichard RK, Gazzinelli A, Grant WN, Boatin BA, McCarthy JS, et al A research agenda for helminth diseases of humans: the problem of helminthiases. PLoS Negl Trop Dis. 2012;6(4):e1582 Epub 2012/05/01. 10.1371/journal.pntd.0001582 22545164 PMC3335854

[21] Prichard RK, Basanez MG, Boatin BA, McCarthy JS, Garcia HH, Yang GJ, et al A research agenda for helminth diseases of humans: intervention for control and elimination. PLoS Negl Trop Dis. 2012;6(4):e1549 Epub 2012/05/01. 10.1371/journal.pntd.0001549 22545163 PMC3335868

[22] Molyneux DH, Bradley M, Hoerauf A, Kyelem D, Taylor MJ. Mass drug treatment for lymphatic filariasis and onchocerciasis. Trends Parasitol. 2003;19(11):516–22. Epub 2003/10/29. . 14580963 10.1016/j.pt.2003.09.004

[23] Turner HC, Walker M, Churcher TS, Osei-Atweneboana MY, Biritwum NK, Hopkins A, et al Reaching the London Declaration on neglected tropical diseases goals for onchocerciasis: an economic evaluation of increasing the frequency of ivermectin treatment in Africa. Clin Infect Dis. 2014;59(7):923–32. Epub 2014/06/20. 10.1093/cid/ciu467 24944228 PMC4166981

[24] Turner HC, Churcher TS, Walker M, Osei-Atweneboana MY, Prichard RK, Basanez MG. Uncertainty surrounding projections of the long-term impact of ivermectin treatment on human onchocerciasis. PLoS Negl Trop Dis. 2013;7(4):e2169 Epub 2013/05/02. 10.1371/journal.pntd.0002169 23634234 PMC3636241

[25] Awadzi K, Boakye DA, Edwards G, Opoku NO, Attah SK, Osei-Atweneboana MY, et al An investigation of persistent microfilaridermias despite multiple treatments with ivermectin, in two onchocerciasis-endemic foci in Ghana. Ann Trop Med Parasitol. 2004;98(3):231–49. Epub 2004/05/04. 10.1179/000349804225003253 . 15119969

[26] Mackenzie CD, Geary TG. Flubendazole: a candidate macrofilaricide for lymphatic filariasis and onchocerciasis field programs. Expert Rev Anti Infect Ther. 2011;9(5):497–501. Epub 2011/05/26. 10.1586/eri.11.30 . 21609260

[27] Ceballos L, Mackenzie C, Geary T, Alvarez L, Lanusse C. Exploring the potential of flubendazole in filariasis control: evaluation of the systemic exposure for different pharmaceutical preparations. PLoS Negl Trop Dis. 2014;8(5):e2838 Epub 2014/05/31. 10.1371/journal.pntd.0002838 24874646 PMC4038472

[28] Longo M, Zanoncelli S, Messina M, Scandale I, Mackenzie C, Geary T, et al *In vivo* preliminary investigations of the effects of the benzimidazole anthelmintic drug flubendazole on rat embryos and fetuses. Reprod Toxicol. 2014;49:33–42. Epub 2014/07/06. 10.1016/j.reprotox.2014.06.009 . 24994687

[29] Ash LR, Riley JM. Development of *Brugia pahangi* in the jird, *Meriones unguiculatus*, with notes on infections in other rodents. J Parasitol. 1970;56(5):962–8. Epub 1970/10/01. . 4396173

[30] Weil GJ, Chandrashekar R, Liftis F, McVay CS, Bosshardt SC, Klei TR. Circulating parasite antigen in *Brugia pahangi-*infected jirds. J Parasitol. 1990;76(1):78–84. Epub 1990/02/01. . 2405144

[31] Denham DA, Suswillo RR, Rogers R, McGreevy PB. Studies with *Brugia pahangi* 17. The anthelmintic effects of diethylcarbamazine. J Parasitol. 1978;64(3):463–8. Epub 1978/06/01. . 26737

[32] Hübner MP, Ehrens A, Koschel M, Dubben B, Lenz F, Frohberger SJ, et al Macrofilaricidal efficacy of single and repeated oral and subcutaneous doses of flubendazole in *Litomosoides sigmodontis* infected jirds. PLoS Negl Trop Dis. In Press. 10.1371/journal.pntd.0006320 PMC6334906 30650105

[33] Sjoberg H, Pionnier N, Aljayyoussi G, Metuge HM, Njouendou AJ, Chunda VC, et al Short-course, oral flubendazole does not mediate significant efficacy against *Onchocerca* adult male worms or *Brugia* microfilariae in murine infection models. PLoS Negl Trop Dis. In Press. 10.1371/journal.pntd.0006356 PMC6334903 30650071

[34] Voronin D, Cook DA, Steven A, Taylor MJ. Autophagy regulates *Wolbachia* populations across diverse symbiotic associations. Proc Natl Acad Sci USA. 2012;109(25):E1638–46. Epub 2012/05/31. 10.1073/pnas.1203519109 22645363 PMC3382551

[35] Voronin D, Bachu S, Shlossman M, Unnasch TR, Ghedin E, Lustigman S. Glucose and glycogen metabolism in *Brugia malayi* is associated with *Wolbachia* symbiont fitness. PloS One. 2016;11(4):e0153812 Epub 2016/04/15. 10.1371/journal.pone.0153812 27078260 PMC4831766

[36] Franz M, Zahner H, Benten P. Fine-structure alterations in female *Brugia malayi* and *Litomosoides carinii* after *in vivo* treatment with flubendazole. Parasitol Res. 1990;76(5):401–5. Epub 1990/01/01. . 2352917 10.1007/BF00933547

[37] O'Neill M, Geary JF, Agnew DW, Mackenzie CD, Geary TG. *In vitro* flubendazole-induced damage to vital tissues in adult females of the filarial nematode *Brugia malayi*. Int J Parasitol Drugs Drug Resist. 2015;5(3):135–40. 10.1016/j.ijpddr.2015.06.002 26288741 PMC4534755

[38] O’Neill M, Mansour A, DiCosty U, Geary J, Dzimianski M, McCall SD, et al An *in vitro/in vivo* model to analyze the effects of flubendazole exposure on adult female *Brugia malayi*. PLoS Negl Trop Dis. 2016;10(5):e0004698 10.1371/journal.pntd.0004698 27145083 PMC4856366

[39] Vialpando M, Smulders S, Bone S, Jager C, Vodak D, Van Speybroeck M, et al Evaluation of three amorphous drug delivery technologies to improve the oral absorption of flubendazole. J Pharm Sci. 2016;105(9):2782–93. Epub 2016/04/27. 10.1016/j.xphs.2016.03.003 27113473 PMC4988473

[40] Tweats DJ, Johnson GE, Scandale I, Whitwell J, Evans DB. Genotoxicity of flubendazole and its metabolites *in vitro* and the impact of a new formulation on *in vivo* aneugenicity. Mutagenesis. 2016;31(3):309–21. Epub 2015/10/08. 10.1093/mutage/gev070 26443851 PMC4840262

[41] Fuchs R. Summary of evaluations performed by the Joint FAO/WHO Expert Committee on Food Additives: Flubendazole: International Programme on Chemical Safety; 2001 [cited 2018]. Available from: http://www.inchem.org/documents/jecfa/jecmono/v31je02.htm.

