# Supplementary material for: Efficacy of subcutaneous doses and a new oral amorphous solid dispersion formulation of flubendazole on male jirds (Meriones unguiculatus) infected with the filarial nematode Brugia pahangi
Source: PLoS Negl Trop Dis. 2019 Jan 16;13(1):e0006787. doi: 10.1371/journal.pntd.0006787 (PMC6334909; doi:10.1371/journal.pntd.0006787)
Supplement: S1 Table — Jirds received 10 mg/kg subcutaneous (SC) flubendazole injections once or for 5 days or oral (PO) doses of 0.2, 0.6, 1.5, 6 or 15 mg/kg flubendazole for 5 consecutive days. (PDF) [file pntd.0006787.s001.pdf]

SUPPLEMENTARY TABLE 1

Expt 1 ASD FBZ 68 days Takedown	Total # of adult worms per jird						# of female worms per jird						# microfilariae per jird					
Animal	No tx	FBZ 10mpk SC x 1d	FBZ 10mpk SC x 5d	ASD FBZ 0.2mpk PO x 5d	ASD FBZ 0.6mpk PO x 5d	ASD FBZ 1.5mpk PO x 5d	No tx	FBZ 10mpk SC x 1d	FBZ 10mpk SC x 5d	ASD FBZ 0.2mpk PO x 5d	ASD FBZ 0.6mpk PO x 5d	ASD FBZ 1.5mpk PO x 5d	No tx	FBZ 10mpk SC x 1d	FBZ 10mpk SC x 5d	ASD FBZ 0.2mpk PO x 5d	ASD FBZ 0.6mpk PO x 5d	ASD FBZ 1.5mpk PO x 5d
1	48	48	0	16	1	10	22	27	0	2	0	6	5,000,000	260,000	0	7,440,000	840,000	206,667
2	54	11	0	39	1	83	28	0	0	27	0	46	4,440,000	133,333	0	1,400,000	240,000	1,220,000
3	71	11	0	3	1	2	44	0	0	0	0	1	7,080,000	1,700,000	0	0	120,000	913,333
4	63	3	0	52	11	122	44	0	0	33	1	64	5,780,000	740,000	0	2,526,667	400,000	5,440,000
5	23	47	0	40	9	1	8	0	0	17	2	0	2,560,000	2,380,000	200,000	3,960,000	780,000	366,685
6	3	4	0	0	88	18	1	2	0	0	33	10	140,000	0	2,913,333	26,667	7,120,000	600,000
7		7		26	38	1		4		15	15	1		1,160,000		1,140,000	1,820,000	500,000
**Geometric means**	30.5	11.3	0	10.0	6.1	8.7	14.6	0.6	0	3.3	1.0	4.0	2,622,892	73,903	20	117,909	717,411	753,423
**Lower 95% CI of geo. mean**	8.6	4.1	0	1.3	1.1	1.4	3.2	0	0	0.3	0	0.5	557,336	276	0	309	206,213	285,840
**Upper 95% CI of geo. mean**	107.6	31.0	0	80.0	34.4	55.3	67.0	5.0	0	35.6	9.6	34.3	12,343,640	19,815,050	110,684	44,981,410	2,495,860	1,985,891
**% Reduction compared to no tx group**	-	63%	100%	67%	80%	72%	-	96%	100%	77%	93%	72%	-	97%	100%	96%	73%	71%
**Passes Shapiro-Wilk normality test?**	N too small	No	N too small	Yes	No	No	N too small	No	N too small	Yes	No	No	N too small	Yes	N too small	Yes	No	No
**Significance**		No	Yes	No	No	No		No	Yes	No	No	No		No	Yes	No	No	No
**P Value from Prism**		> 0.999	0.001	> 0.999	0.519	> 0.999		0.098	0.003	> 0.999	0.264	> 0.999		0.234	0.008	> 0.999	0.596	0.596

Expt 2 ASD FBZ 72 days Takedown	Total # of adult worms per jird				# of female worms per jird				# microfilariae per jird			
Animal	No tx	FBZ 10mpk SC x 1d	ASD FBZ 6mpk PO x 5d	ASD FBZ 15mpk PO x 5d	No tx	FBZ 10mpk SC x 1d	ASD FBZ 6mpk PO x 5d	ASD FBZ 15mpk PO x 5d	No tx	FBZ 10mpk SC x 1d	ASD FBZ 6mpk PO x 5d	ASD FBZ 15mpk PO x 5d
1	4	14	5	9	4	0	0	1	2,400,000	100	23,200	900,000
2	47	13	37	*	20	0	15	*	5,300,000	320,000	300,000	*
3	31	15	78	88	5	0	34	40	4,100,000	210,000	4,500,000	2,200,000
4	74	50	12	7	26	23	0	4	3,500,000	950,000	500,000	1,200,000
5	13	6	4	41	8	2	0	8	1,300,000	1,720,000	600,000	2,500,000
6	73	12	10	24	31	0	0	10	1,100,000	800,000	600,000	3,000,000
7	30	7	44	9	8	0	28	4	1,500,000	1,000,000	5,600,000	130,000
8	37	23	42	4	22	0	19	0	8,200,000	2,000,000	2,200,000	200,000
9	56	37	45	30	36	0	17	16	6,600,000	6,400,000	1,600,000	2,300,000
10	13	12	5	20	0	0	0	3	1,300,000	400,000	400,000	3,000,000
**Geometric means**	28.3	15.3	17.6	17.0	8.3	0.2	1.5	4.0	2,779,756	367,535	732,468	1,137,840
**Lower 95% CI of geo. mean**	14.6	9.5	8.0	8.0	2.4	0.1	0.2	1.0	1,634,860	41,505	236,003	457,940
**Upper 95% CI of geo. mean**	54.8	24.6	39.0	36.0	29.0	0.9	11.2	15.1	4,726,427	3,254,565	2,273,317	2,827,179
**% Reduction compared to no tx group**	-	46%	38%	40%	-	97%	82%	52%	-	87%	74%	59%
**Passes Shapiro-Wilk normality test?**	Log10 transformed data passed normality test, significance determined using Log10 transformed data with Ordinary One-Way ANOVA followed by Holm-Sidak Multiple Comparison test				Yes	No	No	No	Yes	No	No	Yes
**Significance**	-	No	No	No	-	Yes	No	No	-	Yes	No	No
**P Value from Prism**	-	0.383	0.424	0.424	-	0.005	0.455	> 0.999	-	0.028	0.072	0.375

* Animal euthanized due to injury
