# Supplementary material for: Efficacy of subcutaneous doses and a new oral amorphous solid dispersion formulation of flubendazole on male jirds (Meriones unguiculatus) infected with the filarial nematode Brugia pahangi
Source: PLoS Negl Trop Dis. 2019 Jan 16;13(1):e0006787. doi: 10.1371/journal.pntd.0006787 (PMC6334909; doi:10.1371/journal.pntd.0006787)

Supplementary Table 2

Chemical structures of JNJ-161941 (FBZ) and the metabolites JNJ-114699 (H-FBZ) and JNJ-1809600 (R-FBZ)

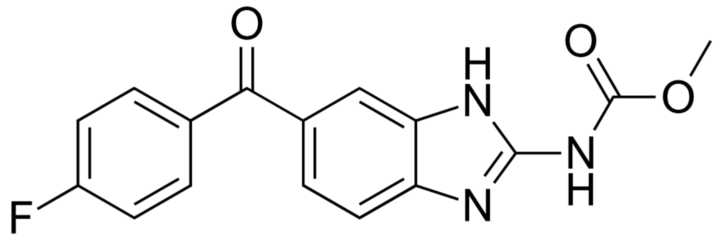

Flubendazole (FBZ) JNJ-161941

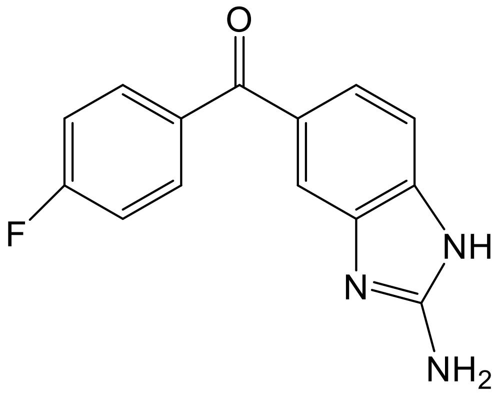

Hydrolyzed Flubendazole (H-FBZ) JNJ-114699

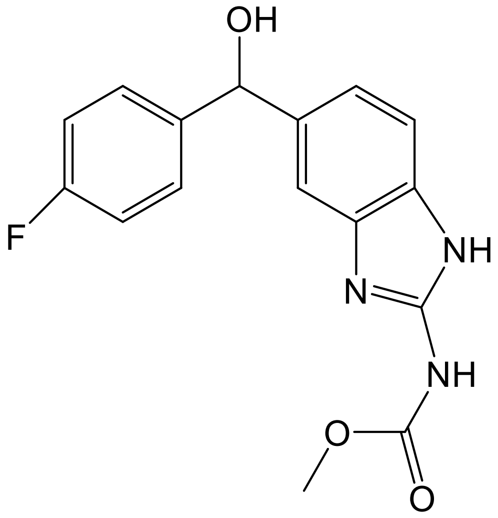

Reduced Flubendazole (R-FBZ) JNJ-1809600

Plasma (ng/ml) concentrations of JNJ-161941 (FBZ) and the metabolites JNJ-114699 (H-FBZ) and JNJ-1809600 (R-FBZ) after oral (PO) and single or repeated subcutaneous (SC) administration to male jirds (n = 2 or 3 per time point).

Experiment 1

FBZ JNJ-161941

G1		SD 10 mg/kg SC		n=2
day	hr			mean
1	1	3.38	3.74	3.56
1	3	4.88	6.11	5.50
1	8	7.86	4.27	6.07
1	24	2.30	3.49	2.90
1	168	1.45	3.39	2.42
1	336	2.42	1.08	1.75
1	504	0.716	1.56	1.14
1	672	0.769	1.80	1.28
1	840	1.30	2.09	1.70
1	1008	1.50	0.962	1.23
1	1176	0.755	0.638	0.70
1	1344	1.06	1.39	1.23
1	1512	0.797	1.13	0.96

H-FBZ JNJ-114699

G1		SD 10 mg/kg SC		n=2
day	hr			mean
1	1	1.67	2.19	1.93
1	3	6.40	3.83	5.12
1	8	4.24	4.11	4.18
1	24	1.98	4.83	3.41
1	168	2.31	2.26	2.29
1	336	1.43	0.42	0.92
1	504	BLQ	1.98	1.98
1	672	1.130	0.85	0.99
1	840	BQL	1.42	1.42
1	1008	1.24	1.590	1.42
1	1176	1.050	BLQ	1.05
1	1344	BQL	1.02	1.02
1	1512	0.515	0.84	0.68

R-FBZ JNJ-1809600

G1		SD 10 mg/kg SC		n=2
day	hr			mean
1	1	0.28	0.24	0.26
1	3	0.92	0.92	0.92
1	8	1.49	1.21	1.35
1	24	0.36	0.37	0.37
1	168	0.32	0.63	0.47
1	336	0.42	0.38	0.40
1	504	0.207	BLQ	0.10
1	672	BLQ	0.35	0.18
1	840	0.30	0.48	0.39
1	1008	0.27	0.000	0.13
1	1176	BQL	BQL	BQL
1	1344	BQL	BQL	0.30
1	1512	0.217	0.000	0.11

G2		5x 10 mg/kg SC		n=2
day	hr			mean
1	2	8.18	6.73	7.46
2	2	12.3	14.9	13.6
3	2	10.9	13.9	12.4
4	2	14.5	9.98	12.2
5	1	23.2	25.3	24.3
5	3	16.3	25.0	20.7
5	8	17.0	17.6	17.3
5	24	24.9	27.3	26.1
5	48	9.03	17.5	13.3
5	216	11.0	6.27	8.64
5	384	6.95	12.7	9.83
5	552	7.05	12.7	9.88
5	720	9.24	6.10	7.67
5	888	8.52	8.37	8.45
5	1056	4.84	10.7	7.77
5	1224	8.00	4.50	6.25
5	1392	6.13	4.07	5.10

G2		5x 10 mg/kg SC		n=2
day	hr			mean
1	2	5.84	4.59	5.22
2	2	13.0	13.9	13.5
3	2	12.2	18.0	15.1
4	2	18.4	16.5	17.5
5	1	22.0	28.9	25.5
5	3	18.0	26.1	22.1
5	8	17.0	18.5	17.8
5	24	24.1	33.5	28.8
5	48	15.5	19.0	17.3
5	216	14.0	13.1	13.6
5	384	12.4	15.7	14.1
5	552	9.89	20.0	14.9
5	720	12.6	10.0	11.3
5	888	11.3	10.5	10.9
5	1056	8.48	15.2	11.8
5	1224	10.2	8.28	9.24
5	1392	8.74	3.56	6.15

G2		5x 10 mg/kg SC		n=2
day	hr			mean
1	2	0.766	0.767	0.767
2	2	1.99	1.77	1.88
3	2	1.33	1.74	1.54
4	2	1.91	1.18	1.55
5	1	2.63	2.61	2.62
5	3	1.93	3.12	2.53
5	8	2.12	1.94	2.03
5	24	3.76	2.62	3.19
5	48	1.05	3.25	2.15
5	216	1.85	1.36	1.61
5	384	1.16	2.64	1.90
5	552	0.859	2.88	1.87
5	720	0.873	1.14	1.01
5	888	1.41	1.85	1.63
5	1056	0.751	2.63	1.69
5	1224	0.844	0.979	0.912
5	1392	0.806	0.577	0.692

G3		5x 0.2 mg/kg PO			n=3	
Day	hr				mean	stdev
1	2	14.1	2.82	5.37	7.43	5.92
2	2	11.7	14.0	3.60	9.77	5.46
3	2	13.2	8.82	8.68	10.2	2.6
4	2	25.0	13.2	4.44	14.2	10.3
5	2	13.9	14.8	15.2	14.6	0.7
5	24	1.01	0.353	0.315	0.559	0.391

G3		5x 0.2 mg/kg PO			n=3	
Day	hr				mean	stdev
1	2	14.1	11.7	8.06	11.3	3.0
2	2	13.1	21.6	13.6	16.1	4.8
3	2	14.4	17.3	12.8	14.8	2.3
4	2	23.8	22.5	16.2	20.8	4.1
5	2	17.7	16.1	20.1	18.0	2.0
5	24	3.26	2.03	2.02	2.44	0.71

G3		5x 0.2 mg/kg PO			n=3	
Day	hr				mean	stdev
1	2	6.10	0.543	1.88	2.84	2.90
2	2	2.11	3.25	0.603	1.99	1.33
3	2	5.06	1.41	2.07	2.85	1.95
4	2	4.64	1.87	0.656	2.39	2.04
5	2	4.58	2.62	2.68	3.29	1.11
5	24	BQL	BQL	BQL		

G4		5x 0.6 mg/kg PO			n=3	
Day	hr				mean	stdev
1	2	17.5	40.3	7.45	21.8	16.8
2	2	11.3	27.6	28.2	22.4	9.6
3	2	30.9	68.6	7.84	35.8	30.7
4	2	9.21	52.2	25.9	29.1	21.7
5	2	26.0	46.1	43.3	38.5	10.9
5	24	0.319	0.557	0.764	0.547	0.223

G4		5x 0.6 mg/kg PO			n=3	
Day	hr				mean	stdev
1	2	40.3	38.1	33.5	37.3	3.5
2	2	57.8	67.5	60.4	61.9	5.0
3	2	48.7	56.5	48.4	51.2	4.6
4	2	62.8	60.5	72.0	65.1	6.1
5	2	71.9	40.2	79.2	63.8	20.7
5	24	3.78	6.67	5.56	5.34	1.46

G4		5x 0.6 mg/kg PO			n=3	
Day	hr				mean	stdev
1	2	3.52	16.9	1.41	7.28	8.40
2	2	4.35	5.45	4.06	4.62	0.73
3	2	4.31	22.1	0.909	9.11	11.4
4	2	2.50	6.38	3.27	4.05	2.05
5	2	4.09	12.2	5.20	7.16	4.40
5	24	BQL	BQL	BQL		

G5		5x 1.5 mg/kg PO			n=3	
Day	hr				mean	stdev
1	2	81.2	84.4	41.0	68.9	24.2
2	2	31.7	91.9	115	79.5	43.0
3	2	50.0	66.6	53.9	56.8	8.7
4	2	54.8	111	45.3	70.4	35.5
5	2	119	97.0	37.7	84.6	42.1
5	24	0.702	0.823	2.60	1.38	1.06

G5		5x 1.5 mg/kg PO			n=3	
Day	hr				mean	stdev
1	2	106	76.5	108	96.8	17.6
2	2	283	126	178	196	80
3	2	133	177	139	150	24
4	2	302	128	129	186	100
5	2	188	285	158	210	66
5	24	17.8	9.50	27.3	18.2	8.9

G5		5x 1.5 mg/kg PO			n=3	
Day	hr				mean	stdev
1	2	4.33	16.1	7.70	9.38	6.06
2	2	4.74	30.8	20.0	18.5	13.1
3	2	2.26	9.92	9.87	7.35	4.41
4	2	8.63	32.2	10.1	17.0	13.2
5	2	5.00	14.7	5.20	8.30	5.54
5	24	BLQ	BLQ	0.567	0.19	0.33

Experiment 2

FBZ

G1		SD 10 mg/kg SC		
Day	Hr	sample1	sample2	mean
1	1	6.77	5.11	5.94
1	3	6.89	9.19	8.04
1	8	3.38	6.9	5.14
1	24	5.88	4.26	5.07
1	48	3.99	2.57	3.28
7	168	3.00	2.13	2.57
21	504	2.50	1.45	1.98
35	840	1.38	123	62.2
49	1176	1.63	1.37	1.50
63	1512	0.595	0.706	0.651

G2		5x 6 mg/kg PO		
Day	Hr	sample1	sample2	mean
1	2	176	361	268
2	2	500	428	464
3	2	289	448	368
4	2	464	383	424
5	1	868	668	768
5	2	408	464	436
5	4	245	171	208
5	8	39.3	63.6	51.5
5	24	1.02	6.05	3.54

G3		5x 15 mg/kg PO		
Day	Hr	sample1	sample2	mean
1	2	704	2420	1562
2	2	2480	1688	2084
3	2	2628	2368	2498
4	2	1240	708	974
5	1	1196	2112	1654
5	2	884	2568	1726
5	4	928	648	788
5	8	366	290	328
5	24	32.7	45.5	39.1

H-FBZ

G1		SD 10 mg/kg SC		
Day	Hr	sample1	sample2	mean
1	1	2.37	2.38	2.375
1	3	3.38	7.11	5.245
1	8	3.47	6.67	5.07
1	24	7.35	5.1	6.225
1	48	4.9	2.95	3.925
7	168	2.19	2.11	2.15
21	504	1.77	1.98	1.875
35	840	1.77	6.21	3.99
49	1176	1.69	1.63	1.66
63	1512	1.68	1.13	1.405

G2		5x 6 mg/kg PO		
Day	Hr	sample1	sample2	mean
1	2	263	236	250
2	2	172	266	219
3	2	245	492	368
4	2	636	464	550
5	1	380	612	496
5	2	412	428	420
5	4	218	153	185
5	8	123	187	155
5	24	13.9	51.0	32.5

G3		5x 15 mg/kg PO		
Day	Hr	sample1	sample2	mean
1	2	572	1008	790
2	2	1040	932	986
3	2	1028	868	948
4	2	912	728	820
5	1	696	872	784
5	2	568	892	730
5	4	744	568	656
5	8	722	309	516
5	24	143	137	140

R-FBZ

G1		SD 10 mg/kg SC		
Day	Hr	sample1	sample2	mean
1	1	BQL	BQL	
1	3	1.12	0.733	0.9265
1	8	BQL	0.812	0.406
1	24	BQL	BQL	
1	48	BQL	BQL	
7	168	BQL	BQL	
21	504	BQL	BQL	
35	840	BQL	BQL	
49	1176	BQL	BQL	
63	1512	BQL	BQL	

BQL set to zero in order to calculate the mean

G2		5x 6 mg/kg PO		
Day	Hr	sample1	sample2	mean
1	2	27.5	34.7	31.1
2	2	40.8	81.6	61.2
3	2	40.8	47.2	44
4	2	28.2	38.5	33.4
5	1	109	92.8	101
5	2	46.8	41.2	44
5	4	35.8	37.2	36.5
5	8	5.82	11.6	8.71
5	24	BQL	0.947	0.474

BQL set to zero in order to calculate the mean

G3		5x 15 mg/kg PO		
Day	Hr	sample1	sample2	mean
1	2	55.6	192	124
2	2	241	127	184
3	2	194	279	237
4	2	159	59.6	109
5	1	83.6	176	130
5	2	47.6	166	107
5	4	109	69.2	89.2
5	8	46.8	46.9	46.9
5	24	4.84	4.31	4.58

Plasma concentrations of FBZ, H-FBZ and R-FBZ 2 hours after dose administration with repeated oral treatment of male jirds with 0.2 – 15 mg/kg ASD FBZ.

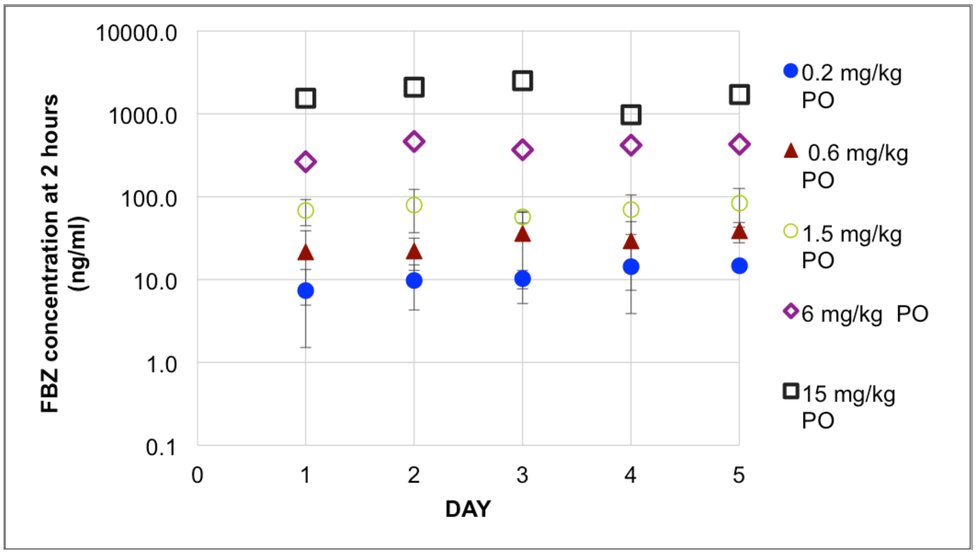

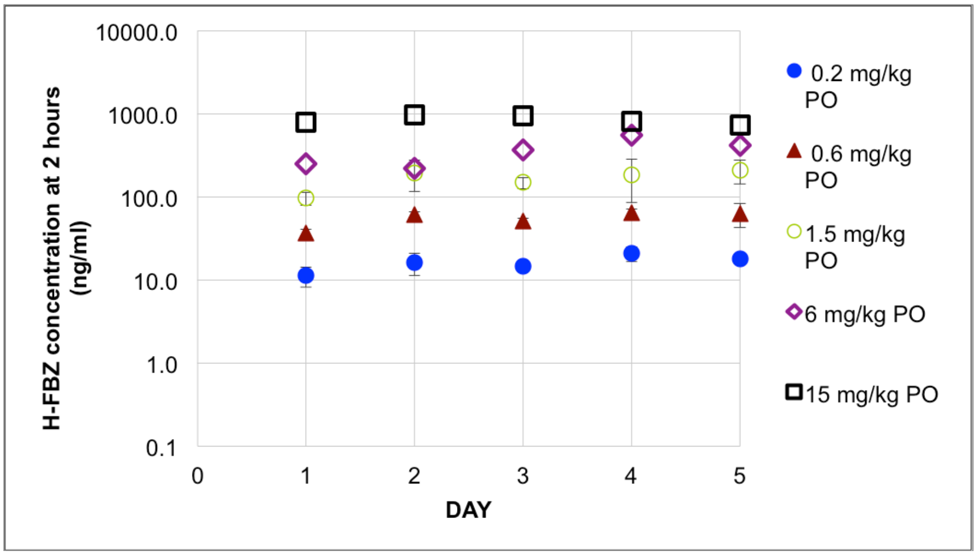

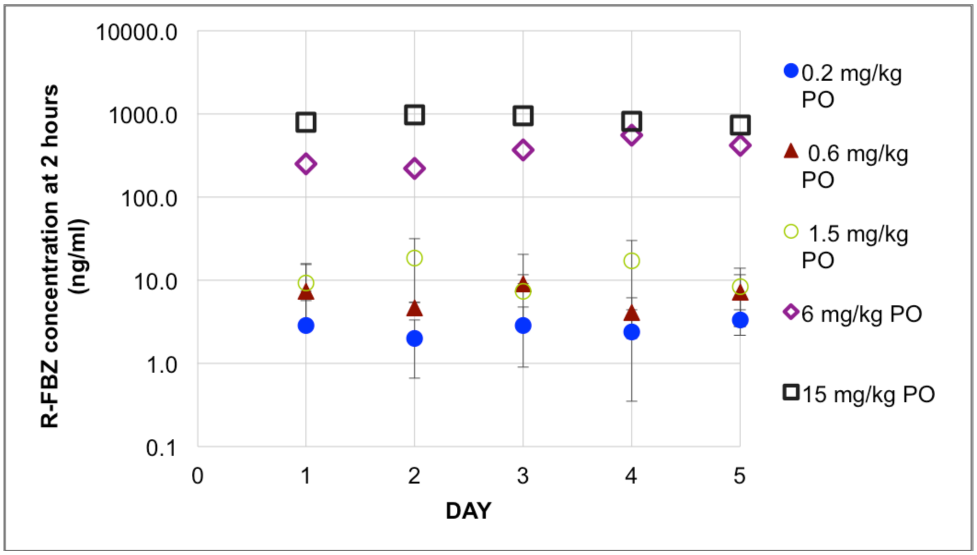

Supplement: S2 Table — (PDF) [file pntd.0006787.s002.pdf]
